# A comparative study of the bacterial diversity and composition of nursery piglets’ oral fluid, feces, and housing environment

**DOI:** 10.1038/s41598-024-54269-5

**Published:** 2024-02-19

**Authors:** Vinicius Buiatte, Ana Fonseca, Paloma Alonso Madureira, Andréia Cristina Nakashima Vaz, Polyana Cristine Tizioto, Ana Maria Centola Vidal, Erika Ganda, Vera Letticie de Azevedo Ruiz

**Affiliations:** 1 Department of Animal Science, College of Agricultural Sciences, The Pennsylvania State University, University Park, PA 16802 USA; 2 Department of Veterinary Medicine, Faculty of Animal Science and Food Engineering, Universidade de São Paulo, Pirassununga, SP Brazil; 3 NGS Genomic Solutions, Piracicaba, SP Brazil

**Keywords:** Swine, Saliva, Feces, 16S rRNA gene, Microbiome, Microbiology, Microbial communities, Environmental microbiology, Infectious-disease diagnostics

## Abstract

The oral cavity is the portal of entry for many microorganisms that affect swine, and the swine oral fluid has been used as a specimen for the diagnosis of several infectious diseases. The oral microbiota has been shown to play important roles in humans, such as protection against non-indigenous bacteria. In swine, studies that have investigated the microbial composition of the oral cavity of pigs are scarce. This study aimed to characterize the oral fluid microbiota of weaned pigs from five commercial farms in Brazil and compare it to their respective fecal and environmental microbiotas. Bacterial compositions were determined by 16S rRNA gene sequencing and analyzed in R Studio. Oral fluid samples were significantly less diverse (alpha diversity) than pen floor and fecal samples (*P* < 0.01). Alpha diversity changed among farms in oral fluid and pen floor samples, but no differences were observed in fecal samples. Permutational ANOVA revealed that beta diversity was significantly different among sample types (*P* = 0.001) and farms (*P* = 0.001), with separation of sample types (feces, pen floor, and oral fluid) on the principal coordinates analysis. Most counts obtained from oral fluid samples were classified as Firmicutes (80.4%) and Proteobacteria (7.7%). The genera *Streptococcus*, members of the *Pasteurellaceae* family, and *Veillonella* were differentially abundant in oral fluid samples when compared to fecal samples, in which *Streptococcus* was identified as a core genus that was strongly correlated (SparCC) with other taxa. Firmicutes and Bacteroidota were the most relatively abundant phyla identified in fecal and pen floor samples, and *Prevotella_*9 was the most classified genus. No differentially abundant taxa were identified when comparing fecal samples and pen floor samples. We concluded that under the conditions of our study, the oral fluid microbiota of weaned piglets is different (beta diversity) and less diverse (alpha diversity) than the fecal and environmental microbiotas. Several differentially abundant taxa were identified in the oral fluid samples, and some have been described as important colonizers of the oral cavity in human microbiome studies. Further understanding of the relationship between the oral fluid microbiota and swine is necessary and would create opportunities for the development of innovative solutions that target the microbiota to improve swine health and production.

## Introduction

Microbiota is the assemblage of microorganisms present in a defined environment that live in a symbiotic relationship with the host^1^. Advances in genetic sequencing and bioinformatics allowed scientists to further explore the composition and function of this community of microorganisms that play important roles in health, nutrition, development, and physiology, especially of those that inhabit the gastrointestinal tract (GIT)^2^.

In swine, several factors can affect the gut microbial composition, function, and relationship with the host, such as diet, age, diseases, management factors, animal breed, water sources, and antimicrobial usage^3,4^. In addition, aspects intrinsic to experimental methodologies may influence differences in microbiome data among studies, such as location of the GIT sampled (small vs. large intestines), specimen (intestinal content vs. mucosa), sequencing-associated factors, and bioinformatic tools used for analyses^5^.

The microbiome development in the GIT of piglets mainly begins at birth, through exposure to maternal (vaginal mucosa, skin, colostrum, and feces) and environmental bacteria (floor and equipment)^6,7^. The diversity of the GIT microbiome in swine significantly increases after birth, up to the early breeder or finisher stages, as early colonizers change the gut microenvironment, facilitating the colonization of strict anaerobes^8^. Firmicutes and Bacteroidota usually account for most of the phyla found in swine fecal samples, wherein *Prevotella* has been commonly reported to be the most abundant genus^4^. Longitudinal characterization of the swine microbiome shows *Prevotella* as the most abundant bacteria in fecal samples between 10 to 13 weeks of age, decreasing after 16 weeks of age. Conversely, the proportion of *Anaerobacter* spp. may increase after 22 weeks of age and become more abundant. Other genera, such as *Lactobacillus*, *Fusobacterium*, *Oscillospira*, *Escherichia*, *Roseburia*, *Faecalibacterium*, and *Bacteroides* are also part of the dominant gut microbiota of pigs during the grower-finisher period^9^.

Weaning is a very stressful period for piglets because of the separation from the sow, the mix of litters, and the change to solid-based diets^10,11^. These changes cause a shift in the bacterial community by decreasing Firmicutes and increasing Bacteroidota, in which *Prevotella* becomes the dominant genus post-weaning^12–14^. Changes in microbial composition occur rapidly, with significant shifts in bacterial community structure as little as four days after weaning^15^.

The swine fecal microbiome has been extensively characterized, but little is known about the oral microbiome. The oral cavity is the portal of entry for an array of microorganisms, including bacteria, archaea, protozoa, and viruses. This complex community is distributed into niches within the oral cavity, performing several metabolic functions that contributes to the host’s homeostasis^16^.

In humans, the oral microbiota has been well-characterized, and more than 700 taxa have been identified, such as *Streptococcus* spp. and *Veillonella* spp.^16,17^. It has been shown that the oral microbiome can influence microbiomes in other parts of the body. For instance, the oral microbiota influences colonization of the respiratory tract and protects the host against pathogens^18^. However, dysbiosis in the oral cavity can trigger important oral and systemic diseases, through the translocation of opportunistic bacteria or chronic inflammation^16^.

Studies that characterized the oral microbiome in animals are more focused on species commonly affected by oral diseases, such as dogs, cats, horses, and cattle^19^. However, the oral microbiome has been linked to important animal diseases, such as bovine respiratory disease (BRD), where researchers have shown the presence of common BRD pathogens in the oral microbiota of calves^20^. In pigs, the identification of *Streptococcus suis* as part of the oral cavity microbiota has shown a potential link to the disease that causes septicemia in post-weaned piglets^21,22^.

The swine oral fluid, a mixture of saliva and mucosal transudate, is a reliable alternative biological sample for screening several diseases, such as Porcine Reproductive Respiratory Syndrome Virus (PRRSV), *Mycoplasma hyopneumoniae*, Influenza, and Porcine circovirus 2 (PCV2). Microorganisms, antibodies, and other metabolites in the oral fluid can be used to better understand the oral microbiome and its importance in swine health^23^. Yet, few studies have characterized the swine oral fluid microbiota and its role in homeostasis^22,24,25^.

Given that the oral cavity is the portal of entry for many microorganisms, characterizing the oral fluid microbiota of weaned piglets can provide information for epidemiological studies of diseases that challenge the swine industry, as well as contribute to topics in gastrointestinal health and microbiome studies. Therefore, the objectives of our study were to determine the oral fluid microbiota of healthy nursery pigs from commercial farms in Brazil and compare its composition to fecal and environmental microbiotas.

## Results

### Sequencing data

A total of 8,457,658 raw reads were generated for all samples (Feces: 4,023,619 reads; Pen floor: 692,101 reads; Oral fluid: 3,741,938 reads). Bacterial 16S rRNA amplicon sequencing yielded a total of 3,528,897 reads after preprocessing (Feces: 34,154.6 reads/sample ± 8232.3; Oral fluid: 31,028.9 reads/sample ± 5268.2; Pen floor: 30,387.2 reads/sample ± 11,480.6) encompassing a total of 5592 taxa in the final dataset. The raw sequences are available at PRJNA880285.

### Taxonomic composition

**Figure 1:**
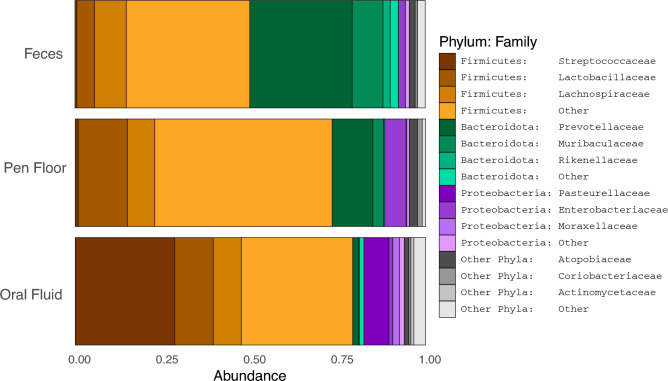
Average relative abundance (%) of Phylum:Family levels identified in the feces, pen floor and oral fluid of nursery pigs raised in five commercial swine farms in Brazil.

At the phylum level, Firmicutes was highly relatively abundant in all sample types (feces, pen floor and oral fluid) (Fig. 1). Fecal samples and pen floor samples shared the three most relatively abundant phyla (Firmicutes, Bacteroidota and Proteobacteria; > 90%), whereas in the oral fluid samples, the three most relatively abundant phyla were Firmicutes (80.4%; 20.6%), followed by Proteobacteria (7.7%; 15.4%) and Actinobacteroita (3.2%; 3.0%), respectively. The highest relative abundance of Firmicutes was obtained in oral fluid samples (80.4%; 20.6%) followed by pen floor samples (77.0%; 25.4%), and fecal samples (49.7%; 16.9%). Bacteroidota was mostly identified in fecal samples (40.3%; 13.9%), followed by pen floor samples (14.2%; 10.7%) and oral fluid samples (2.1%; 3.2%). The highest relative abundance of Proteobacteria was identified in oral fluid samples (7.7%; 15.4%), followed by pen floor samples (1.1%; 1.6%) and fecal samples (0.42%; 1.8%).

At the family level, the three most relatively abundant families found in fecal samples were *Prevotellaceae* (29.7%; 9.8%), representing the phylum Bacteroidota, followed by *Lachnospiraceae* (8.0%; 6.9%), from the phylum Firmicutes, and *Muribaculaceae* (7.1%; 7.8%) from the phylum Bacteroidota. In pen floor samples, the three most relatively abundant families were *Lactobacillaceae* (7.9%; 11.8%), *Ruminococcaceae* (12.13%; 6.1%), both from the phylum Firmicutes, and *Prevotellaceae* (10.4%; 9.3%), representing the phylum Bacteroidota*.* In oral fluid samples, the three most relatively abundant families were *Streptococcaceae* (24.7%; 27.7%), *Lactobacillaceae* (8.2%; 9.1%), and *Veillonellaceae* (7.3%; 5.4%), all representing the phylum Firmicutes.

**Figure 2:**
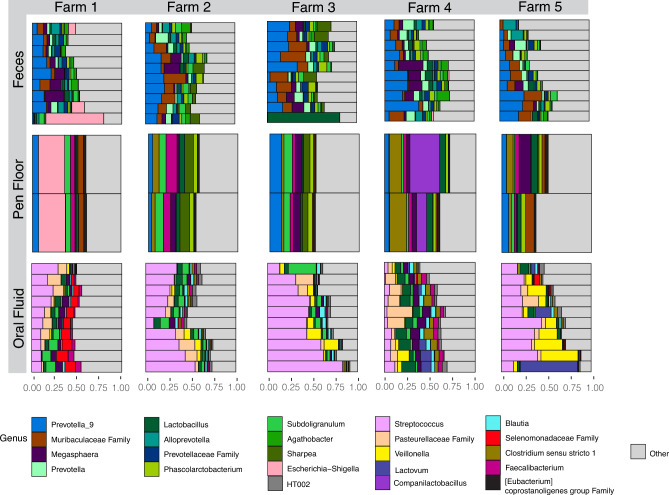
Relative abundance (%) of genera identified in the feces, pen floor and oral fluid of nursery pigs raised in five commercial swine farms in Brazil. Each horizontal bar represents one sample. Oral fluid and feces were collected individually (10 animals/farm), and pen floor samples were collected from two pens in each farm. The 12 most abundant genera were used, and the remainder were included as “Other”.

At the genus level, high variability of the taxa among the animals and farms was observed (Fig. 2). Pen floor samples were less variable within the same farm, showing a similar relative abundance of taxa. However, among different farms, pen floor samples were unsurprisingly distinct. From the genera identified, *Prevotella*_9 was the most relatively abundant genus in pen floor (5.8%; 2.5%) and fecal samples (12.7%; 12.3%), whereas *Streptococcus* was the most relatively abundant genus in oral fluid samples (23.3%; 25.5%).

Complete tables with the relative abundances of taxa in each sample at the phylum, family, and genus levels are provided on GitHub (https://github.com/aff30/Pig-Microbiology).

### Alpha diversity

**Figure 3:**
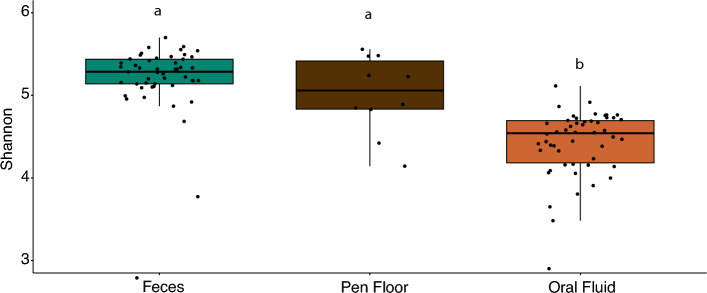
Alpha diversity (Shannon’s index) of microbial communities obtained from feces, pen floor and oral fluid samples collected in five commercial farrow-to-finish farms in Brazil.

**Figure 4:**
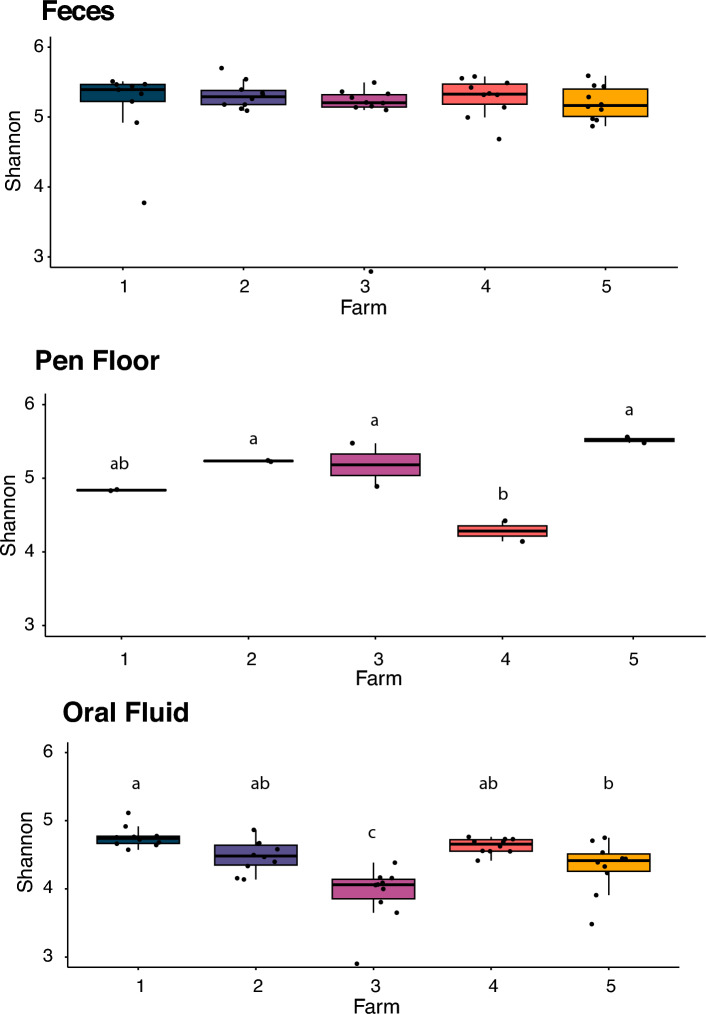
Shannon’s indexes obtained from microbial communities identified in three different sample types (oral fluid, feces, pen floor swabs) collected from nursery pigs raised in five commercial farrow-to-finish farms in Brazil. Different superscripts denote statistical differences (P ≤ 0.05) using Tukey’s HSD test.

Alpha diversity was significantly different among sample types (*P* < 0.01; Fig. 3), in which oral fluid samples had significantly lower alpha diversity than the pen floor and fecal samples (*P* < 0.01). There was no difference in alpha diversity between the pen floor and fecal samples (*P* = 0.444). Shannon indexes were not different among farms for fecal samples (*P* = 0.619), but significantly different in oral fluid (*P* < 0.01) and pen floor samples (*P* = 0.011) (Fig. 4).

### Beta diversity

**Figure 5:**
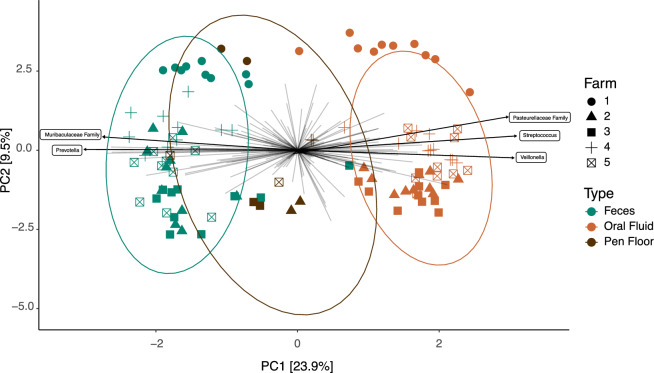
Principal coordinates analysis (PCA) of three sample types (feces, pen floor and oral fluid) collected from nursery pigs raised in five commercial farrow-to-finish farms in Brazil.

The PCA shows differences in the microbial composition among sample types (Fig. 5). Bacterial communities of fecal samples are closer to those of pen floor samples, and more distant from those of oral fluid samples. Separation can also be observed for Farm 1. The five most abundant bacterial taxa loadings are shown in the PCA. The Pasteurellaceae family, the genera *Streptococcus* and *Veillonella* were associated with oral fluid samples, whereas the Muribaculaceae family and the genus *Prevotella* were associated with the fecal samples. Permutational ANOVA showed that sample type (PERMANOVA, *P* = 0.001) and farm (PERMANOVA, *P* = 0.001) had significantly different microbial populations. Post hoc comparisons revealed significant differences between all sample types and farms.

### Differential relative abundance

**Figure 6:**
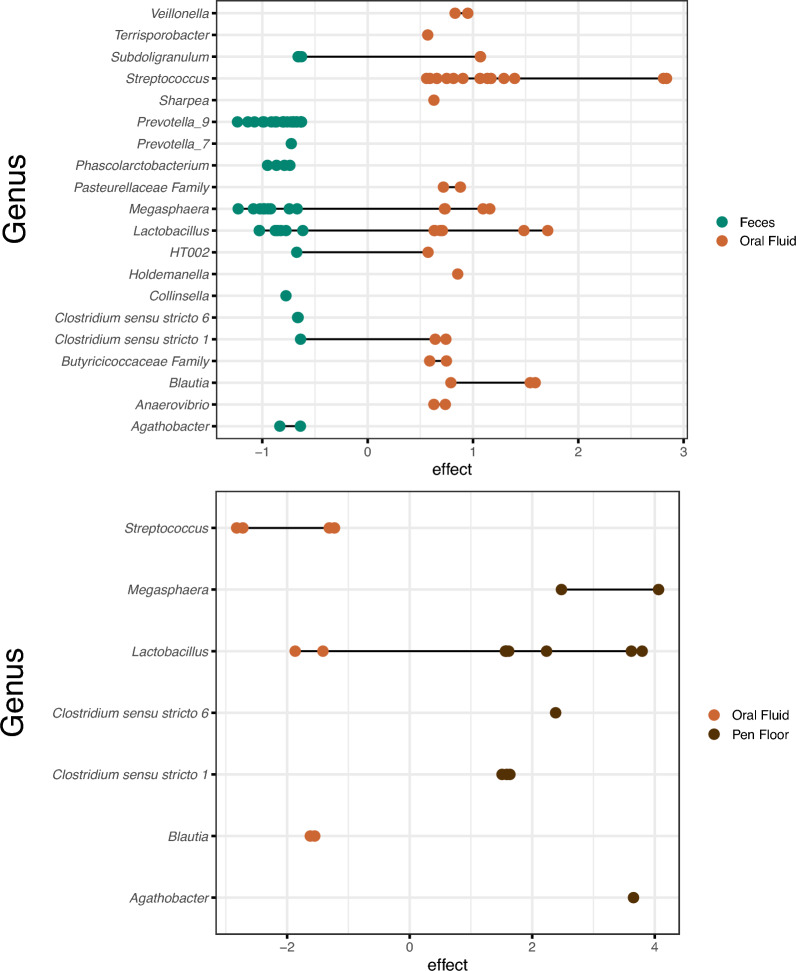
Differentially abundant taxa obtained from center log-ratio transformed data at the genus level, identified in the feces, pen floor or oral fluid of nursery piglets raised in commercial farrow-to-finish farms in Brazil. Each dot represents one amplicon sequence variant (ASV) classified at the genus level.

Several taxa were identified as differentially relatively abundant when comparing two sample types. Differentially abundant taxa identified with ALDEx2 are depicted in Fig. 6. When comparing oral fluid and fecal samples, *Streptococcus*, *Veillonella,* members of the *Pasteurellaceae* family were more differentially abundant in oral fluid samples. *Prevotella_9* and *Prevotella_*7 were more differentially relatively abundant in fecal samples than in oral fluid samples. *Lactobacillus*, *Megasphaera* and *Subdoligranulum* ASVs were differentially abundant in both fecal and oral fluid samples, with a higher abundance of ASVs in fecal samples than in oral fluid samples. *Clostridium *sensu stricto 1 ASVs were more abundant in oral fluid samples than in fecal samples.

When comparing oral fluid and pen floor samples, *Megasphaera*, *Clostridium* (senso stricto 1 and 6) and *Agathobacter* were more differentially relatively abundant in pen floor samples than in oral fluid samples. *Streptococcus* and *Blautia* were more differentially relatively abundant in oral fluid samples. *Lactobacillus* ASVs were differentially abundant in both oral fluid and pen floor samples, with more ASVs differentially abundant in pen floor samples. No significant differences in differential relative abundances were observed between the pen floor and fecal samples (data not shown).

### Network analysis

**Figure 7:**
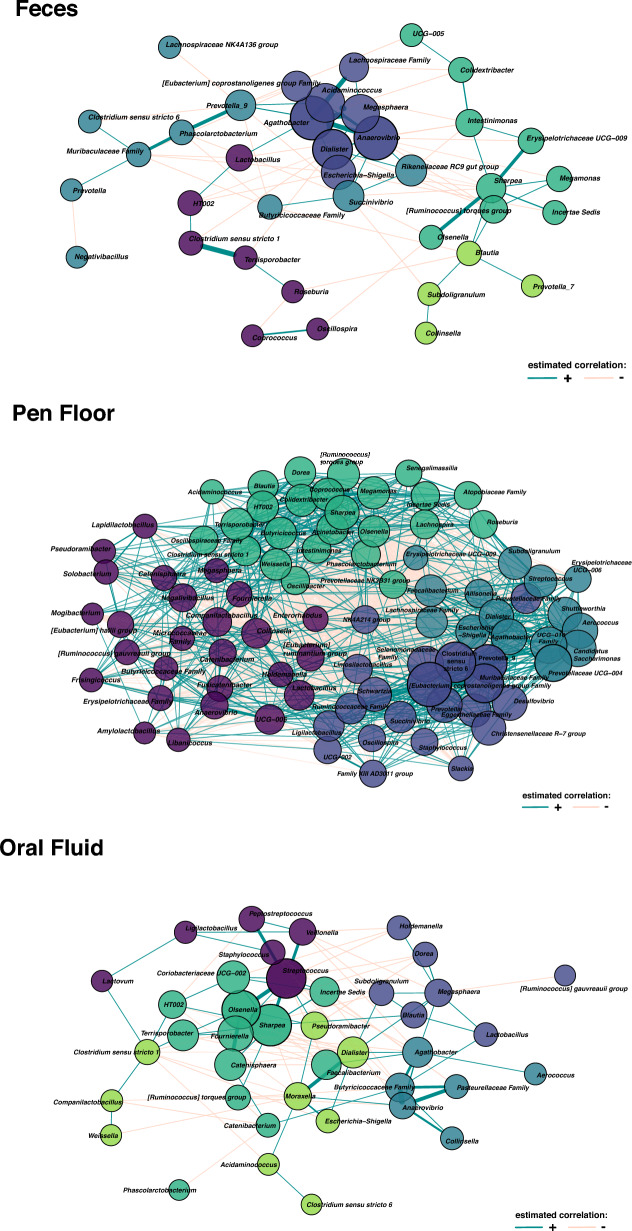
Correlation networks inferred with SparCC (Sparse Correlations for Compositional data) from genera identified in feces, pen floor and oral fluid samples of nursery piglets raised in commercial farrow-to-finish farms in Brazil. Nodes (circles) represent genera, and the size is proportional to the corresponding eigenvector score, in which large circles are well-connected genera. Different node colors represent clusters based on the I-Louvain clustering method. Edges (lines) were formed based on the correlation between the nodes, in which the width represents the magnitude of the correlation based on the eigenvector centrality, and the color indicates whether the correlation is positive (cyan) or negative (light salmon).

Based on the network analysis constructed with Sparce Correlations for Compositional data (SparCC), we observed taxa counts that were positively and negatively correlated, as well as the strength of their connections, as depicted in Fig. 7. A summary of the centrality measures in each sample type is available as supplementary material in our GitHub page (https://github.com/aff30/Pig-Microbiology).

In the oral fluid samples, *Sharpea* and *Streptococcus* obtained the highest degree, betweenness and closeness scores, showing that these were major core taxa within the network. *Streptococcus* had a strong positive correlation with *Veillonella*, *Peptostreptococcus*, *Sharpea*, *Olsenella*, and *Staphylococcus.* A member of the *Pasteurellaceae* Family was positively correlated with *Anaerovibrio*, and a member of the *Butyricicoccaceae* Family, and negatively correlated with *Streptococcus*. In the fecal samples, *Agathobacter*, *Anaerovibrio* and *Dialister* were the major core taxa in the network, in which *Agathobacter* and *Anaerovibrio* had the highest closeness and eigenvector scores. Interestingly, *Prevotella*_7 was not part of the cluster in which *Prevotella* and *Prevotella*_9 formed, showing a difference in the correlation pattern based on different strains or subgroups within the same genus. In the pen floor samples, a very complex network was observed, in which hubs were formed by *Eubacterium coprostanoligenes* group Family, *Candidatus Saccharimonas*, *Clostridium* sensu stricto 6, *Muribaculaceae* Family and *Prevotella*_9 formed. *Clostridium* sensu stricto 6 was the genus with the highest score in all centrality measures used in this analysis.

## Discussion

This study aimed to characterize the microbial composition of the oral fluid of weaned pigs and compare it to the microbial composition of fecal samples and pen floor samples. The swine oral cavity is the portal of entry for many microorganisms of importance in swine production and public health. Moreover, the swine oral fluid has the potential to be used as a biological sample for screening for diseases^23^. The oral fluid is composed of saliva and mucosal transudate that contains water, hormones, antibodies, ions, and several proteins involved in the immune response^26^. Thus, understanding the microbial composition of the oral fluid may open opportunities to study the host-oral microbiota crosstalk and its role in homeostasis and disease.

Studies that describe the swine oral fluid are scarce. To date, only three articles have been published. In Vietnam, 11 weaned piglets were evaluated. Among the 558 OTUs identified, 18 were uniquely found in the oral fluid when compared to other samples (feces, vagina mucus, equipment swabs). The most abundant genera (> 50%) were *Streptococcus*, *Moraxella*, *Actinobacillus,* and *Rothia*^22^*.* Another study explored the microbial community relationships among different samples (feces, air, oral fluid, and trachea) of growing pigs infected with *Mycoplasma hyopneumoniae.* The five most abundant genera identified in the oral fluid samples were *Clostridium*, *Terrisporobacter*, *Phascolarctobacterium*, *Leptotrichia*, and *Streptococcus*^25^. The oral fluid of sows has also been studied, and the most abundant genera were *Lactobacillus*, *Corynebacterium*, *Acinetobacter*, *Staphylococcus*, *Rothia*, *Aerococcus*, and *Clostridium*^24^. In this study, oral fluid samples were mostly comprised of Firmicutes, followed by Proteobacteria and Actinobacteriota. The five most abundant genera obtained in oral fluid samples were *Streptococcus*, followed by members of the *Pasteurellaceae* family, *Veillonella*, *Lactobacillus*, and *Subdoligranulum*.

From the studies published to date, it can be noted that *Streptococcus* is a commonly found genus of the oral fluid. *Streptococcus* are commensal colonizers of the oral cavity throughout the pig’s life^22^, even in diseased pigs^25^. However, dysbiosis of the oral microbiota can lead to an increased population of *S. suis*, a potentially zoonotic pathogen that causes severe septicemia in weaned pigs^21^*.* In a human study, *Streptococcus*, *Veillonella* and *Prevotella* represented almost 50% of the microbial composition of the oral fluid^27^. In humans, *Streptococcus* spp. are considered early colonizers of the oral cavity and produce several metabolic compounds, including bacteriocins, that change the oral microenvironment and guide microbial succession^17^. In the network analysis, *Streptococcus* was one of the most influential genera identified in oral fluid samples, showing a strong positive correlation to *Veilonella*, *Sharpea*, *Olsenella*, and *Peptostreptococcus*. Further research is needed to investigate the role of *Streptococcus* spp. in the swine oral microbiome as it can be an important modulator of other community members, and thus, a key member of the swine oral microbiome.

The *Pasteurellaceae* family was also differentially abundant in oral fluid samples. Members of this family are usually commensal microorganisms of the gastrointestinal tract and upper respiratory tract, but some species are opportunistic pathogens associated with important swine diseases in growing pigs, such as *Pasteurella multocida* and *Glaesserella parasuis* (formerly known as *Haemophilus parasuis*)^28,29^. *P. multocida* has been isolated from pig bite wound infections in humans, and *Pasteurella* spp. are common isolates of bite wound infections from other animal species^30^.

Oral fluid samples were less diverse (alpha diversity) than fecal samples and pen floor samples, which was expected since the oral cavity has specific microenvironmental conditions and less nutrient availability compared to other segments of the gastrointestinal tract^31^. The main source of nutrients for bacteria in the oral cavity is the oral fluid rather than food, and many studies in humans and animals showed evidence that diet is not a major factor that influences oral microbial composition^32^. Compared to fecal samples, oral fluid, and pen floor samples presented a higher variability in alpha diversity among farms. Housing conditions change substantially among farms, due to location, management, personnel, facilities, and many other factors that can impact the microbiota^33^. The oral cavity of swine is in constant contact with the pen floor because pigs have classic exploratory behaviors, such as rooting and sniffing^34^. These factors could be associated with the high variability of alpha diversity in both oral fluid and pen floor samples among farms.

The fecal microbiota of swine has been extensively described because it is easy to collect and allows repeated sampling over time, with comparable results to other sampling methods^35^. However, most studies published to date were conducted in regions where production conditions are very different than in southern countries. In Brazil, pigs are often raised on pen floors in naturally ventilated houses that are often subjected to extreme temperatures and other environmental challenges that can induce changes in microbial composition^36^. To the best of our knowledge, the present study was the first to characterize the microbial composition of the oral fluid, feces, and environment of nursery piglets raised in commercial farrow-to-finish swine farms in Brazil.

Firmicutes, mostly comprised of Gram-positive bacteria, and Bacteroidota, predominantly comprised of Gram-negative bacteria, accounted for over 90% of the taxa identified in fecal samples in this study. In South Korea, researchers have shown Bacteroidota (59.6%-63.1%) and Firmicutes (34.2%-35.8%) as the most abundant phyla in weaned piglets collected at different ages^12,13^. In China, one study monitored 10 pigs from weaning to slaughter to determine the bacterial composition of feces, and the relative abundance of Firmicutes and Bacteroidota accounted for over 90% of the taxa found in samples from piglets at two months of age^37^. Studies in North America and Europe have also shown similar results. In Canada, a study investigating the fecal microbiota of 18 pigs during a full production cycle, showed that weaning piglets had most counts (> 70%) comprising representatives of the phyla Firmicutes and Bacteroidota^38^. In the United States, a longitudinal study with 17 pigs showed that, at 61 days of age, Firmicutes and Bacteroidota accounted for more than 85% of the microbial composition, where Firmicutes was the most abundant phylum, followed by Bacteroidota^11^. In France, a longitudinal study assessed the fecal microbiota of 31 piglets until 70 days of age, and the phyla Firmicutes and Bacteroidota corresponded to more than 90% of the total counts^39^.

Firmicutes and Bacteroidota seem to be the most relatively abundant phyla found in fecal samples, even in studies held in different countries, under different weather conditions, management procedures, feed sources, and antibiotic treatment. The balance of Firmicutes and Bacteroidota is important to observe, especially between growth stages. The abrupt change from a milk-based diet to a solid-based diet has been associated with changes in the ratio Firmicutes:Bacteroidota^40^. Some members of the phylum Bacteroidota, such as *Prevotella*, can produce important enzymes for the digestion of plant cell walls present in the diets^41^. *Prevotella* produce acetate, a substrate used by butyrate-producing bacteria^42^. Butyrate is a short-chain fatty acid (SFCA) that the host can use as a source of energy^43^. In this study, *Prevotella* was the most relatively abundant genus in fecal and environmental samples, which shows that the microbial composition of the animals was more adapted to a plant-based diet. In addition, the network analysis revealed that *Prevotella*_9 was the second genus with the highest degree scores, showing that not only *Prevotella*_9 was relatively abundant, but also is a taxon highly correlated with several other bacteria in fecal samples.

From the 10 most relatively abundant genera identified in pen floor samples and in fecal samples, four common genera were found (*Prevotella*_9, *Megasphera*, *Lactobacillus* and *Subdoligranulum*), but no differentially abundant taxa were identified. Oral fluid and pen floor samples shared four genera among their 10 most relatively abundant taxa (*Lactobacillus*, *Subdoligranulum*, *Megasphera* and *Clostridium* sensu stricto 1). From these genera, *Lactobacillus* and *Clostridium* sensu stricto 1 were more differentially abundant in pen floor samples. There are no studies in the literature that assessed the relationship between the pig’s gut microbiome and the environmental microbiota. In intensive swine production, nursery piglets are often moved to different buildings or farms, and apart from adult animals, which may substantially change the microbial profile of the environment^44^. In this study, we did not evaluate factors that could influence the microbial composition of the pens, such as size, floor type (slatted or concrete), number of animals/pen or cleaning and disinfection procedures. Future studies should explore the influence of the environmental microbiota in swine microbial development and function, since the environment can harbor microorganisms that challenge the host’s homeostasis.

The fecal, oral fluid and environmental samples were clustered differently in the PCA, supporting the expected dissimilarities between the three sample types. Similar observations were reported in a study that determined the beta diversity of fecal samples, saliva samples and swabs from the feeder and drinker, with saliva samples forming a separate cluster in relation to the feces and overlapping samples from feeders and drinkers^22^. The microbial composition analysis of this study illustrated the pathway by which microbes can travel in the fecal–oral route. These environments harbor different microbiotas, and understanding their establishment and influence in the host may help elucidate pathogen transmission, create, and improve different diagnostic tools, as well as solutions that aim to modulate the microbiota to improve animal health and production.

In conclusion, under the conditions of our study, the oral fluid showed a distinct microbiota from fecal and environmental samples, with several differentially abundant taxa that may be important colonizers of the oral cavity, such as *Streptococcus*, that are highly correlated to other taxa in the oral fluid microbiome. The composition of the fecal samples was consistent with other studies and similar among the farms studied, which shows that the nursery piglets’ fecal microbiome is fairly conserved. Based on the evidence shown in human studies, the oral microbiota may play an important role in health and disease. Therefore, future studies should aim to further understand the oral microbiota functions in swine and determine its influence on host’s homeostasis.

## Materials and methods

### Sampling

Fecal and oral fluid samples were collected from healthy nursery piglets (60–70 days old) from five different farrow-to-finish farms in the state of Sao Paulo, Brazil (number of sows: Farm 1 – 37; Farm 2 – 2000; Farm 3 – 1333; Farm 4 – 151, and Farm 5 – 1650). All animals were commercial crossbred pigs weaned at 3 weeks of age and fed a commercial maize-soybean-based diet ad libitum. On each farm, two pens of weaned pigs were randomly chosen, and five animals were randomly selected from each pen for sampling. Fifty animals and ten pens were used in this study. All procedures were previously approved by the Animal Ethics Committee (CEUA) of the Faculty of Animal Science and Food Engineering (FZEA/USP) (Approval n. 9,741,230,818), and were conducted following the norms issued by the National Council for the Control of Animal Experimentation (CONCEA, Brazil).

Fecal and oral fluid samples were collected individually. The oral fluid was collected with sterile collection device made of cotton rolls tied to strings and stored in 15 mL sterile polypropylene centrifuge tubes. The device was offered to the animals for voluntary chewing for one minute and transferred back into the tube^45^. Fecal samples were collected during voluntary fecal elimination into 50-mL sterile polypropylene centrifuge tubes without touching other surfaces to avoid cross-contamination. The pens were sampled using the boot sock method on the pen floor, as previously described^46^. Briefly, two layers of sterile disposable polypropylene shoe covers were worn over the researcher’s boot and dragged over the pen’s floor. The inner layer was used to avoid contamination from the boots and the outer layer was used for downstream analyses. The samples were stored in sterile sampling bags (Whirl–Pak®, Wisconsin, USA) and kept on ice until arrival at the laboratory, where they were stored at − 80 °C.

### 16S rRNA sequencing

The bacterial microbiota of all samples was determined by 16S rRNA sequencing, targeting the V4 region, following the procedures described by Caporaso et al.^47^. First, samples were processed for DNA extraction using the MagMAX™ CORE Mechanical Lysis Module kit (Thermo Fisher™, Waltham, MA, USA). Briefly, 2 g of feces, the whole cotton roll, or a piece of 25 cm^2^ (5 cm × 5 cm) of the disposable polypropylene shoe cover were used for this step. For the negative control, 200 µl of UltraPure™ DNase/RNase-Free Distilled Water (Invitrogen™) was used. Bacterial DNA was extracted with the MagMAX™ CORE Nucleic Acid Purification Kit (Thermo Fisher™, Waltham, MA, USA) following the manufacturer’s instructions. The KingFisher system (Thermo Fisher™, Waltham, MA, USA) was used for extraction. The quality of the extracted DNA was verified by agarose gel electrophoresis and quantified with a spectrophotometer. DNA sequence data were generated using the Illumina™ MiSeq™ paired-end sequencing platform with reads of 2 × 250 base pairs (bp). The library was prepared according to Illumina™ recommendations, which consisted of two PCRs, two purification steps, two agarose gels, quantification, normalization, multiplexing, and library denaturation. PCRs were performed with a denaturation step at 94 °C for 3 min, followed by 35 cycles of amplification at 94 °C for 45 s, 50 °C for 60 s, and 72 °C for 90 s, with a final extension at 72 °C for 10 min. The first PCR was performed for locus-specific amplification using primers flanking the 292-bp V4 region between 515 (5′-GTG YCA GCM GCC GCG GTA A-3′) and 806 bp (5′-GGA CTA CNV GGG TWT CTA AT-3′) and overhang adapters (forward 5′-TCG TCG GCA GCG TCA GAT GTG TAT AAG AGA CAG-3′ and reverse 5′-GTC TCG TGG GCT CGG AGA TGT GTA TAA GAG ACA G-3′). AMPure™ XP beads (Beckman Coulter®, Brea, CA, USA) were used for purification and the generated fragments were assessed by agarose gel electrophoresis. The second PCR was used to add the 96 barcodes (Nextera XT™ Kit, Illumina®, San Diego, CA, USA) followed by additional purification and validation steps. A heterogenic control, PhiX phage (Illumina®, San Diego, CA, USA), was combined with the amplicon pool. Finally, PhiX and library denaturation were performed for sequencing. Negative controls were included alongside the samples during DNA extraction and PCR amplification. The absence of laboratory contamination was confirmed by absence of bands during gel electrophoresis of negative controls. Total raw reads per sample type (Feces, Pen floor, Oral fluid) were reported.

### Bioinformatics and statistical analysis

Read quality assessment was performed with the FastQC tool (v. 0.12.1)^48^. After quality control, only the forward reads were chosen for further analysis. The Fastp tool (v. 0.23.1)^49^ was used to process the bacterial 16S rRNA sequences utilizing quality profiling, adapter trimming, read filtering, and base correction. The filtered reads were subsequently dereplicated using the DADA2 (v. 1.18) package in R^50^. Chimeras were removed with the removeBimeraDenovo function, and taxonomy was assigned at the genus level using the SILVA database (v138.1.)^51^. The average reads ± standard deviations per sample type were reported. Amplicon sequence variants (ASVs) were constructed and filtered with the decontam R package^52^. The final dataset was purged of non-bacterial ASVs, ASVs that had no phylum assignment, and ASVs whose overall relative abundance was less than 1e-5. Relative abundances are reported as median and interquartile range.

Beta diversity (between-sample diversity or community structure), alpha diversity (within-sample evenness and/or richness), and differential relative abundance of bacterial genera across sample types were evaluated. The Shannon index was used to quantify alpha diversity. Sample type (oral fluid, feces and pen floor swab) and farm (Farm 1-Farm 5) were considered as fixed factors for the Analysis of Variance (ANOVA). Post hoc comparisons were performed with Tukey’s Honest Significant Difference (HSD) test. The microViz package in R was used to construct principal coordinates analysis (PCA) using center log-ratio (CLR) transformed bacterial community data at the genus level^53^. Differences in beta diversity were evaluated using permutational multivariate ANOVA (PERMANOVA) of Aitchison distances using the Adonis test with 999 permutations. The pairwise.adonis function of the vegan software was used to calculate pairwise comparisons^54^.

Differential relative abundance was analyzed using the ALDEx2 software, applying a t-test on CLR transformed data at the genus level, while accounting for sample type variance. Significance was claimed when the expected Benjamini–Hochberg corrected *P-*values were less than 0.05. ALDEx2 package also generated the expected effect size for the paired sample type evaluated (oral fluid vs. pen floor; and oral fluid vs. feces)^55^. All data visualizations were created using the R packages microViz and ggplot2^53,56^ and edited with Adobe Illustrator (v. 27.7).

Network analysis of the association patterns between taxa was performed to infer ecological correlations among taxa in each sample type (feces, pen floor, and oral fluid). Data was preprocessed by filtering out low abundance taxa (< 10 counts) and retaining only those present in at least 20% of the samples. ASVs were agglomerated at the genus level, and the analysis was performed using the NetCoMi (Network Construction and Comparison for Microbiome Data) package (v.1.1.0) in R^57^. Network construction was performed with the Sparce Correlations for Compositional data method (SparCC)^58^ to minimize the occurrence of spurious correlations among taxa. SparCC produces correlation coefficients from CLR-transformed data assuming the large size of the dataset and that the sparsity of the correlations. ASV counts were normalized using CLR, and the *r-*threshold of 0.3 was considered, following the approach of Friedman et al.^58^ The constructed network was analyzed with the netAnalyze function, and genera were clustered with the I-Louvain method^59^. The network analysis output reveals different centrality measures, such as, degree, betweenness, closeness, and eigenvector centralities. Briefly, degree measures the number of connections a node (i.e. genus) maintains. Betweenness refers to nodes acting as bridges, connecting many nodes together. Closeness assesses the proximity of a node to all the other nodes, identifying the central node of the network. Finally, eigenvector centrality measures the number of connections a node has, and the quality of its connections, assigning high scores to nodes connected to well-connected peers.

Data visualization was performed with the plot function, where genera represent nodes, and their respective edges were constructed based on eigenvector centrality. Unconnected nodes were removed, and node size represented the magnitude of the eigenvector score.

## Data Availability

The datasets generated and analyzed in this study can be found on GitHub (https://github.com/aff30/Pig-Microbiology). Raw sequences are available in NCBI, accession number PRJNA880285.
